# Quantum Fisher Information-Guided Diffusion Refinement for Fuzzy and Uncertain Surveillance Regions

**DOI:** 10.3390/e28101151

**Published:** 2026-10-20

**Authors:** Sultan Alam, Piyush Makhija, Abdul Khader Jilani Saudagar, Mubarak Albathan

**Affiliations:** 1 School of Computer Science and Engineering, Galgotias University, Greater Noida 203201, India; sultan.alam23@gmail.com; 2 School of Computing Science and Engineering, VIT Bhopal University, Sehore 466114, India; piyushmakhija26@gmail.com; 3 Information Systems Department, College of Computer and Information Sciences, Imam Mohammad Ibn Saud Islamic University (IMSIU), Riyadh 11432, Saudi Arabia; aksaudagar@imamu.edu.sa; 4 Department of Computer Science, College of Computer Engineering and Sciences, Prince Sattam bin Abdulaziz University, Al-Kharj 16278, Saudi Arabia

**Keywords:** quantum Fisher information, latent diffusion models, image restoration, uncertainty quantification, surveillance imaging

## Abstract

Restoring surveillance images containing both structurally reliable and information-sparse fuzzy regions remains challenging because conventional restoration and diffusion-based methods may either suppress authentic textures or introduce visually plausible but unsupported details. To address this problem, we propose quantum Fisher information-guided diffusion refinement (QFI-Diff), a hybrid framework that incorporates quantum information-theoretic uncertainty into latent diffusion-based image restoration. Specifically, quantum Fisher information (QFI) is employed as a local sensitivity measure to construct a reliability map that distinguishes information-rich regions from uncertain and degraded regions. This map guides the reverse diffusion process by constraining reliable regions while permitting adaptive stochastic refinement in uncertain areas. We establish theoretical guarantees for the proposed framework, including asymptotic convergence of the associated stochastic differential equation, information-theoretic bounds based on the quantum Cramér–Rao bound, computational complexity bounds, and discrete restoration error bounds. Experiments on the DarkFace and RESIDE datasets demonstrate that QFI-Diff achieves competitive structural and perceptual restoration performance compared with the evaluated baselines, as reflected in the SSIM, LPIPS, and FID metrics. These results highlight the potential of quantum information-theoretic uncertainty measures for reliability-aware diffusion-based image restoration.

## 1. Introduction

The ubiquitous deployment of surveillance infrastructure in smart cities, transport hubs, and critical facilities has created a massive influx of visual data. While the volume of data is immense, its utility is frequently compromised by the physical limitations of acquisition [1]. In forensic investigations, critical footage is often captured under the worst-case scenarios: grainy night-vision feeds, foggy perimeter cameras, or shaky, long-range drone footage. The restoration of such imagery is not merely an aesthetic concern; it is a matter of public safety and legal integrity, requiring rigorous validation as seen in clinical domains [2].

The central problem in restoring such imagery is the heterogeneity of degradation. Unlike standard denoising benchmarks where noise is uniform, a real surveillance frame is spectrally diverse. It might contain a perfectly legible license plate in one corner, representing a high signal-to-noise ratio (SNR), while a suspect’s face in the shadows is reduced to a fuzzy, indeterminate blob of pixels. This heterogeneity creates a paradox for automated enhancement systems, which we term the Surveillance Fidelity Paradox. If a system is tuned to aggressively hallucinate high-frequency details to resolve a face, it risks generating artifacts that may not exist, such as altering a facial feature or changing a character on a license plate. Conversely, if the system is restricted to conservative denoising, it prioritizes signal preservation but often results in severe texture wiping in low-light conditions.

Deep learning approaches, particularly convolutional neural networks (CNNs) like LLNet [3] and SRResNet [4], have advanced the state of the art. However, standard supervised learning models are fundamentally regression models. They are typically trained to minimize pixel-wise error metrics such as mean squared error (MSE). By mathematically optimizing for the conditional mean of a multimodal distribution of possible high-resolution images, these networks inherently produce blurry results. If a low-resolution patch could plausibly represent hair, skin, or fabric, the regression solution is the average of all three, resulting in a smooth, waxy texture. This lack of high-frequency variance is catastrophic for forensic analysis, where edge sharpness defines the distinctiveness of a feature, a limitation that persists even in recent transformer-based approaches like SwinIR [5] and Restormer [6].

Generative diffusion models [7,8] have emerged as a powerful alternative. By learning the gradients of the underlying data distribution, diffusion models can sample from the full posterior distribution rather than predicting a single blurred mean. This allows them to synthesize highly realistic high-frequency details, as demonstrated by frameworks like SDEdit [9] and Palette [10]. However, this stochasticity introduces the fatal flaw of hallucination. In regions of high uncertainty, such as heavily degraded shadows, the model relies entirely on its learned priors to fill in missing details. It acts as a dreaming machine, synthesizing statistically probable but factually incorrect features. In a security context, a restoration algorithm cannot be allowed to dream; it must remain anchored to the ground truth observation, a challenge often addressed via formal uncertainty quantification [11].

To solve the Surveillance Fidelity Paradox, we need a mechanism to quantify reliability at a highly granular level—a map that dictates exactly where the generative model is permitted to use its prior in fuzzy regions, and where it must be strictly constrained by the evidence in clear regions. To achieve this, we turn to quantum information theory (QIT). In the field of quantum metrology, quantum Fisher information (QFI) is used to bound the precision with which a parameter can be estimated. Unlike classical variance, which merely measures the spread of intensity values and is easily confused by heavy sensor noise, QFI measures the geometric sensitivity of a state to structural transformations [12,13]. We postulate that by mapping classical image patches to quantum states, QFI can serve as a hyper-sensitive sensor for local information content, distinguishing between structurally invariant stochastic noise and structurally sensitive semantic edges with far greater precision than classical edge detectors.

**Figure 1:**
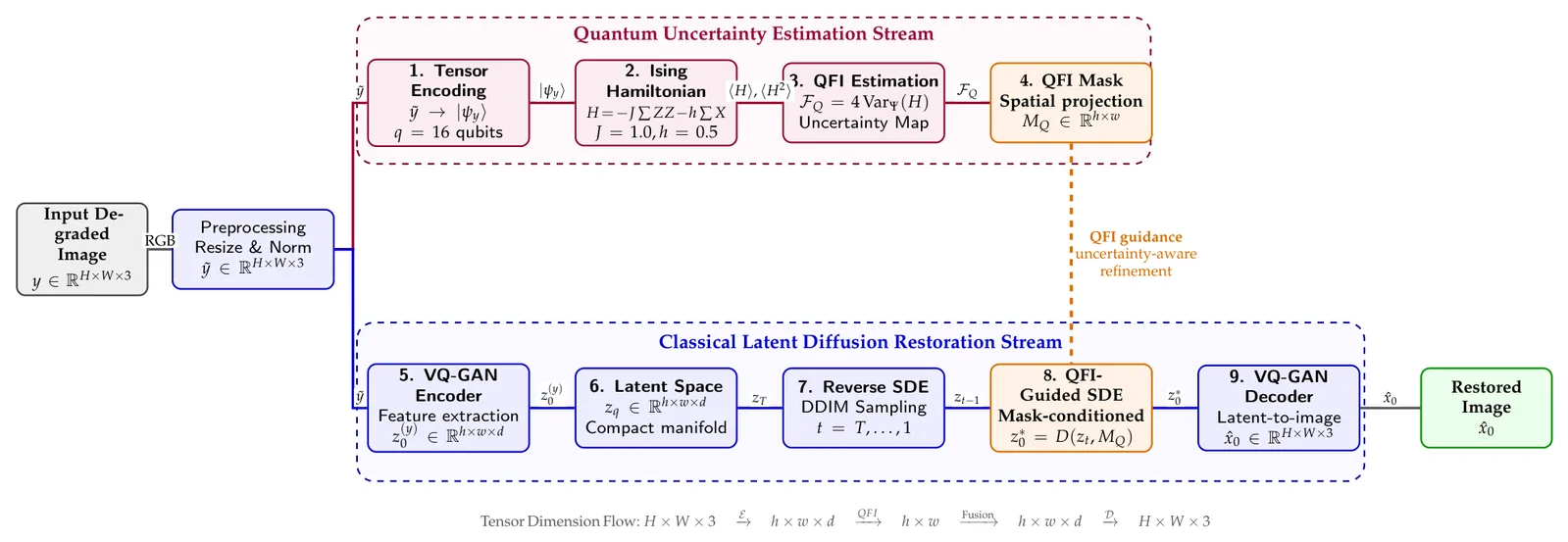
Detailed step-by-step architecture of the proposed QFI-Diff framework. The input image is processed through parallel quantum uncertainty estimation and classical latent restoration streams.

In this paper, we propose QFI-Diff, a unified framework for robust image restoration that merges quantum uncertainty estimation with generative diffusion models. To advance accountable artificial intelligence for surveillance restoration, we prioritize theoretical grounding alongside empirical evaluation, establishing a mathematically grounded mechanism for constraining generative details according to estimated information reliability. Figure 1 illustrates the proposed QFI-Diff architecture. The framework operates in two synergistic streams. The quantum uncertainty stream encodes image patches into probability amplitudes to measure state sensitivity against an Ising Hamiltonian, yielding a deterministic reliability mask *M*. Simultaneously, the classical generative stream processes the image in the latent space. The QFI mask modulates the reverse SDE, enforcing strict evidence consistency in high-QFI regions while permitting generative synthesis in low-QFI (fuzzy) regions. The principal contribution of QFI-Diff is a reliability-guided diffusion restoration mechanism in which a QFI-derived spatial sensitivity measure is used to distinguish regions containing stronger structural evidence from regions with greater uncertainty. The present implementation is quantum-inspired rather than quantum hardware-based. QFI and the associated Ising operator are evaluated using classical tensor network contraction, while the restoration process is performed using a classical latent diffusion model.

## 2. Related Work

To mathematically ground the challenge of surveillance restoration, we must first define the physical degradation model of the sensors. While academic benchmarks often assume additive white Gaussian noise (AWGN), real surveillance sensors exhibit signal-dependent noise. We utilize the Poisson–Gaussian noise model which accurately reflects photon arrival statistics (shot noise) and electronic readout noise. The observed image $y\in R^{H\times W\times C}$ is related to the latent clean image $x_{0}$ by

(1) $$y∼P\left(\alpha x_{0}\right)+N\left(0,\sigma^{2}\right)$$

where P denotes a Poisson distribution and α is the sensor gain. For mathematical tractability in the diffusion context, this is often approximated by a heteroscedastic Gaussian model:

(2) $$y=A\left(x_{0}\right)+\eta ,\;\eta ∼N\left(0,\Sigma \left(x_{0}\right)\right)$$

where $\Sigma \left(x_{0}\right)=\alpha x_{0}+\sigma^{2}$. This dependency of noise variance on signal intensity implies that bright regions are noisier than dark regions in absolute terms but have higher SNR. Dark regions (common in surveillance) are dominated by the constant readout noise $\sigma^{2}$ and have very low SNR. The inverse problem is to recover $x_{0}$ from *y*. This is ill-posed because the operator A is singular or near-singular (information loss), meaning there are infinitely many $x_{0}$ that could result in *y*.

To formalize how this degradation behaves in a generative context, we derive the score function for the Poisson noise case y∼Pois(x). The likelihood is given by the product of individual pixel probabilities:

(3) $$p\left(y|x\right)=\prod_{i}\frac{x_{i}^{y_{i}}e^{-x_{i}}}{y_{i}!}$$

Taking the natural logarithm to find the log-likelihood:

(4) $$\log p\left(y|x\right)=\sum_{i}\left(y_{i}\log x_{i}-x_{i}-\log\left(y_{i}!\right)\right)$$

The score function is the gradient of the log-likelihood with respect to the latent image *x*. Calculating the partial derivative with respect to $x_{i}$:

(5) $$\frac{\partial }{\partial x_{i}}\log p\left(y|x\right)=\frac{y_{i}}{x_{i}}-1$$

Thus, the vector score is

(6) $$\nabla_{x}\log p\left(y|x\right)=\frac{y}{x}-1$$

In diffusion models, since we add Gaussian noise during the forward process, we effectively smooth this discrete Poisson distribution into a continuous Gaussian approximation N(x,x), justifying our Gaussian noise model assumption in Equation (2). This approximation is critical for applying standard diffusion score-matching objectives [8].

To resolve the ill-posedness of this inverse problem, we rely on a prior distribution p(x) learned by a diffusion model, utilizing the score-based generative modeling framework. The forward diffusion process is defined by the Itô stochastic differential equation (SDE) that progressively perturbs data $x_{0}∼p_{data}$ into noise over time t∈[0,T] [7,8]:

(7) dx=f(x,t)dt+g(t)dw

where f(x,t) is the drift coefficient, g(t) is the diffusion coefficient, and *w* is the standard Wiener process. The reverse-time SDE, which generates samples from the prior distribution $p_{0}\left(x\right)$ starting from noise $x_{T}∼N\left(0,I\right)$, is given by

(8) $$dx=\left[f\left(x,t\right)-g^{2}\left(t\right)\nabla_{x}\log p_{t}\left(x\right)\right]dt+g\left(t\right)d\bar{w}$$

where $\bar{w}$ is a standard Wiener process in reverse time, and $\nabla_{x}\log p_{t}\left(x\right)$ is the score function. In practice, a neural network $s_{\theta }\left(x,t\right)$ is trained to approximate this score via denoising score matching:

(9) $$\min_{\theta }E_{t,x_{0},\epsilon }\left[{s_{\theta }\left(x_{t},t\right)-\nabla_{x_{t}}\log p\left(x_{t}|x_{0}\right)}^{2}\right]$$

Our core objective is to modify this reverse SDE in Equation (8) to include a guidance term derived from the observation *y*. We seek to sample from the posterior $p(x_{0}|y)$. Using Bayes’ rule, the score of the posterior is

(10) $$\nabla_{x}\log p_{t}\left(x|y\right)=\nabla_{x}\log p_{t}\left(x\right)+\nabla_{x}\log p_{t}\left(y|x\right)$$

The critical challenge lies in the term $\nabla_{x}\log p_{t}\left(y|x\right)$, which is the likelihood score. In regions where the degradation is severe (high uncertainty), this likelihood is uninformative or actively misleading. We must therefore define a reliability mask $M\left(y\right)\in {\left[0,1\right]}^{H\times W}$ to adaptively weight the likelihood score based on local certainty:

(11) $$\hat{s}\left(x,t\right)=s_{\theta }\left(x,t\right)+\left(1-M\right)\cdot \nabla_{x}\log p_{t}\left(y|x\right)$$

This formulation ensures that in reliable regions (M→0), we strictly follow the measurement evidence, while in fuzzy regions (M→1), we safely rely on the generative prior.

Understanding how to construct this reliability mask *M* requires examining how the field has historically approached image restoration and uncertainty quantification. Early variational approaches treated restoration as an optimization problem with hand-crafted priors. Total Variation (TV) minimization [14] assumed images were piecewise smooth, minimizing ∫|∇I|dx. While effective for denoising, TV methods tend to create staircase artifacts, flattening subtle textures. In low-light enhancement, the dark channel prior (DCP) [15] and Retinex theory assume that illumination changes slowly while reflectance changes quickly. These assumptions often break down in complex surveillance scenes with multiple light sources, leading to halo artifacts and color distortion. Deep learning subsequently introduced end-to-end mapping, with LLNet [3] pioneering stacked autoencoders and the UNet architecture becoming the backbone for restoration. More recently, vision Transformers (ViTs) like SwinIR [5] and Restormer [6] have replaced CNNs due to their ability to model long-range dependencies. However, all these discriminative methods suffer from the limitations of the $L_{1}$ or $L_{2}$ loss functions. As discussed in [4], the pixel-wise average of possible solutions results in perceptual blur. While generative adversarial networks (GANs) attempt to solve this via a discriminator loss, they suffer from mode collapse and training instability, often fabricating textures semantically inconsistent with the ground truth.

To prevent such hallucinations, knowing exactly *where* a model is uncertain is crucial for safety-critical AI [16]. Bayesian neural networks (BNNs) attempt this by placing distributions over network weights p(w|D), utilizing approximations like Monte Carlo (MC) dropout [17]. However, MC dropout requires multiple forward passes, increasing latency linearly, and its resulting uncertainty map often highlights simple object boundaries (aleatoric uncertainty) rather than true information sparsity. Predictive approaches, such as evidential deep learning [18] and conformal prediction [19], output a learned variance map σ(x) alongside the prediction μ(x). The critique of these methods is that the variance structures are learned parameters. If the model is overconfident on out-of-distribution surveillance data, it may predict low variance even in degraded regions [16].

Diffusion models have revolutionized generative modeling, and recent work has rapidly adapted them for inverse problems to address these limitations. Iterative latent variable refinement (ILVR) [20] proposed conditioning the diffusion process by injecting low-frequency information from a reference image, though it fails to handle spatially heterogeneous noise. RePaint [21] adapted diffusion for inpainting by masking the reverse steps ($x_{t-1}=M⊙x_{ground\_truth}+\left(1-M\right)⊙x_{generated}$), but this requires a hard binary mask manually provided by the user. Diffusion posterior sampling (DPS) [22] uses gradient guidance from a measurement operator ∇logp(y|x) but is computationally expensive as it requires backpropagating through the measurement operator at every timestep. Similarly, denoising diffusion restoration models (DDRM) [23] use singular value decomposition (SVD), and denoising diffusion null-space models (DDNM) [24] constrain generation to the null space. More recently, DiffPIR [25] and PiGDM [26] proposed plug-and-play methods using pre-trained denoisers, while ResShift [27] and cold diffusion [28] developed efficient shifting and inversion processes. Unlike these methods, our framework requires a continuous, spectrally aware guidance mechanism that modulates the prior versus the likelihood adaptively, without relying on learned, overconfident predictive variance.

Recent studies have demonstrated the effectiveness of diffusion models for image restoration, while also highlighting the possibility of generating irrelevant content when the input is severely degraded. Yue et al. [29] proposed a joint conditional diffusion model (JCDM) that incorporates the degraded image and a degradation mask as conditional guidance to constrain the diffusion restoration process. This approach demonstrates the importance of providing degradation-aware information to the generative restoration process. In contrast, the proposed QFI-Diff framework derives its spatial guidance from a quantum Fisher information (QFI)-based local information-sensitivity measure. The resulting QFI-guided map is incorporated into the latent diffusion refinement process to provide spatially adaptive guidance for degraded regions. Therefore, the contribution of the proposed method lies in integrating QFI-based information sensitivity with latent diffusion refinement, rather than in the use of diffusion-based restoration or spatial guidance alone. The quantitative results reported in this work are interpreted within the datasets and evaluation protocols considered in our experiments and are not intended to imply universal superiority over all existing image restoration methods. The comparison of representative restoration paradigms considered in this study is summarized in Table 1.

Because learned predictive uncertainty fails, we propose an *axiomatic* measure derived directly from the signal structure itself, borrowing concepts from entropy-based feature extraction [30,31]. To rigorously define this metric for classical image data, we must establish a theoretical bridge between the Euclidean pixel domain $R^{N}$ and the complex quantum Hilbert space H using an isometric mapping $\phi :R^{N}\to H$. We employ Amplitude Encoding, which maps a vector of *N* continuous values into the probability amplitudes of a system of $n=\lceil \log_{2}N\rceil$ qubits, providing exponential compression in state space dimension.

**Table 1:** Taxonomy of representative image restoration methods considered in this study.

Method	Paradigm	Role
SRGAN [4]	GAN-based	Perceptual restoration
SwinIR [5]	Transformer-based	Image restoration
Stable Diffusion v2.1 [32]	Latent diffusion	Generative prior
RePaint [21]	Mask-guided diffusion	Constrained generation
DiffPIR [25]	Inverse-problem diffusion	Restoration solver
ResShift [27]	Diffusion restoration	Image restoration
JCDM [29]	Conditional diffusion	Degradation-aware guidance
QFI-Diff (Ours)	QFI-guided diffusion	Reliability-aware restoration

**Definition** **1** (Amplitude Encoding)**.** *Let $x\in R^{N}$ be a flattened vector representing a local image patch P of size $\sqrt{N}\times \sqrt{N}$. We first apply an $L_{2}$ normalization to ensure the vector adheres to the probabilistic conservation law of quantum mechanics:*

(12) $$\hat{x}=\frac{x}{x_{2}}=\frac{x}{\sqrt{\sum_{i=1}^{N}{\left|x_{i}\right|}^{2}}}$$

*The corresponding quantum state $\psi_{x}$ is constructed as a superposition in the computational basis:*

(13) $$\psi_{x}=\sum_{i=0}^{N-1}{\hat{x}}_{i}i$$

*where {i} represents the orthonormal basis states {00…0,…,11…1}.*

**Remark** **1.** *The normalization condition $\psi_{x}\psi_{x}=1$ implies that we lose the absolute intensity information (luminance) of the patch. However, this is advantageous for our purpose: QFI becomes a measure of relative structural contrast (texture), invariant to global illumination changes. This robustness to lighting conditions is a key advantage over raw gradient magnitude.*

With the visual data mapped to a robust quantum state, we can derive the quantum Fisher information (QFI), the quantum analogue of the classical Fisher information (CFI), which bounds the precision of parameter estimation in quantum metrology.

**Definition** **2.** *Consider a family of quantum states $\rho_{\theta }$ parameterized by a scalar θ. The QFI $F_{Q}\left(\theta \right)$ is defined as*

(14) $$F_{Q}\left(\theta \right)=\left(\rho_{\theta }L_{\theta }^{2}\right)$$

*where $L_{\theta }$ is the symmetric logarithmic derivative (SLD), the Hermitian operator uniquely defined by the Lyapunov equation:*

(15) $$\frac{\partial \rho_{\theta }}{\partial \theta }=\frac{1}{2}\left\{L_{\theta },\rho_{\theta }\right\}=\frac{1}{2}\left(L_{\theta }\rho_{\theta }+\rho_{\theta }L_{\theta }\right)$$

For our application, we are interested in the sensitivity of a pure state ψ to a unitary transformation generated by a Hamiltonian operator *H*. Let the parameter θ represent the strength of a hypothetical perturbation (e.g., a shift or rotation in feature space). The evolved state is

(16) $$\psi_{\theta }=e^{-iH\theta }\psi_{0}$$

Using the Schrödinger equation, the derivative is

(17) $$\partial_{\theta }\psi =-iHe^{-iH\theta }\psi_{0}=-iH\psi_{\theta }$$

For pure states, the QFI formula simplifies significantly. We substitute the state vector derivative into the general pure-state QFI equation:

(18) $$F_{Q}=4\left(\partial_{\theta }\psi \partial_{\theta }\psi -{\left|\psi \partial_{\theta }\psi \right|}^{2}\right)$$

Evaluating the first term:

(19) $$\partial_{\theta }\psi \partial_{\theta }\psi =\psi \left(iH^{†}\right)\left(-iH\right)\psi =\psi H^{2}\psi$$

Evaluating the second term (the fidelity overlap):

(20) $$\psi \partial_{\theta }\psi =\psi \left(-iH\right)\psi =-i\psi H\psi$$

The squared magnitude is

(21) $${\left|-i\psi H\psi \right|}^{2}={\left(\psi H\psi \right)}^{2}$$

Substituting back into Equation (18):

(22) $$F_{Q}=4\left(\psi H^{2}\psi -{\left(\psi H\psi \right)}^{2}\right)$$

Recognizing the term inside the parenthesis as the variance of the observable *H*, we arrive at our fundamental theorem for structural complexity:

(23) $$F_{Q}\left[\psi ,H\right]=4{\mathrm{Var}}_{\psi }\left(H\right)$$

The physical interpretation of this derivation is critical: it proves that the QFI is strictly proportional to the quantum uncertainty (variance) of the generator *H* in the state ψ. If the image patch ψ is an eigenstate of the perturbation *H* (i.e., it is symmetric or invariant under the operation), the variance is zero, and thus QFI is zero. If the patch is highly sensitive to the perturbation (i.e., it has complex structure orthogonal to *H*), the variance is high.

This formulation provides a profound geometric intuition. The space of pure quantum states forms a complex projective space $CP^{d-1}$, where the geometry is defined by the Fubini–Study metric. The Bures distance (or statistical distance) $d_{Bures}$ between two states ρ and ρ+dρ is related directly to the QFI [33]:

(24) $$d_{Bures}^{2}\left(\rho_{\theta },\rho_{\theta +d\theta }\right)=\frac{1}{4}F_{Q}\left(\theta \right)d\theta^{2}$$

This implies that QFI measures the velocity with which the state moves through Hilbert space as the parameter θ changes. High velocity (high QFI) means that the state is traversing a region of high distinguishability—an edge or semantic texture. Low velocity implies the state is stuck in a flat, structural void representing noise or background.

Finally, this geometric perspective bridges our framework to the broader literature of physics-based machine learning. In quantum image processing (QIMP), algorithms like QSobel [34] explore edge detection offering exponential speedups over classical methods. However, most QIMP research assumes the existence of fault-tolerant quantum random access memory (QRAM) [35]. Our work acts as a quantum-inspired bridge: utilizing the formalism of Hilbert spaces and Fisher Information to design superior algorithms that run on classical GPUs [31]. Furthermore, this maps diffusion models to energy-based models (EBMs), where the score function is the gradient of the energy. By mapping pixels to spins, we effectively define a texture energy landscape where regions with high QFI correspond to high-energy domain walls (edges) in the Ising model, connecting our axiomatic reliability mask directly to fundamental thermodynamic stability.

## 3. Methodology: The QFI-Diff Framework

Having established the theoretical optimality of quantum Fisher information as a structural metric, we now introduce our complete end-to-end methodology, the QFI-Diff framework, whose detailed workflow is illustrated in Figure 1.

The core philosophy of this framework is to explicitly separate the quantification of local visual uncertainty from the generative restoration process itself. By establishing a deterministic reliability map first, we create a rigorous mathematical constraint that dictates the behavior of a subsequent classical diffusion model. The process begins with the generation of this pixel-wise reliability mask, which requires transforming classical visual data into a format amenable to quantum measurement.

The primary objective of the initial uncertainty estimation is to compute a scalar uncertainty field M(i,j)∈[0,1] for the degraded input image *Y*. To obtain this field, the input image is divided into overlapping patches $P_{ij}$ of size k×k. Let $N=k^{2}$ denote the number of pixels in each patch, and let $v_{ij}\in R^{N}$ represent its flattened pixel intensities. The normalized pixel vector is defined as

(25) $${\hat{v}}_{ij}=\frac{v_{ij}}{∥v_{ij}{∥}_{2}}.$$

To establish a consistent mapping between classical pixel intensities and the quantum spin representation, we embed this normalized vector into the one-excitation subspace of an *N*-qubit Hilbert space. Specifically, each pixel is associated with a spin-$\frac{1}{2}$ site on a two-dimensional square lattice, and the computational basis state $|e_{a}\rangle$ denotes the configuration in which only the *a*-th spin is excited while all remaining spins are in their ground states. The amplitude-encoded patch is then represented by the *N*-qubit state

(26) $$|\psi_{ij}\rangle =\sum_{a=1}^{N}{\hat{v}}_{ij,a}|e_{a}\rangle ,$$

where

(27) $$\langle e_{a}|e_{b}\rangle =\delta_{ab}.$$

Consequently, the normalization of the pixel vector ensures that $\langle \psi_{ij}|\psi_{ij}\rangle =1$. Although the amplitudes are specified by the *N* pixel values, the state is embedded in the full *N*-qubit Hilbert space of dimension $d=2^{N}$, with the initial state restricted to the *N*-dimensional one-excitation subspace. This distinction establishes a mathematically consistent correspondence between amplitude encoding and the spin-lattice representation.

Once the patch has been embedded into the spin-lattice Hilbert space, its structural sensitivity is evaluated using the transverse-field Ising Hamiltonian. The *N* qubits are arranged according to the original k×k spatial grid, with nearest-neighbor interactions between sites in the cardinal directions. The Hamiltonian is expressed as

(28) $$H_{\mathrm{Ising}}=-J\sum_{\langle a,b\rangle \in E}\sigma_{z}^{\left(a\right)}\sigma_{z}^{\left(b\right)}-h\sum_{a\in V}\sigma_{x}^{\left(a\right)},$$

where *V* denotes the set of lattice sites, *E* denotes the set of nearest-neighbor edges, and $\sigma_{z}^{\left(a\right)}$ and $\sigma_{x}^{\left(a\right)}$ are the Pauli operators acting on the qubit associated with pixel *a*. Each local operator is understood to act as the identity on all other qubits.

The expectation values of the Hamiltonian and its square are evaluated with respect to the embedded state $|\psi_{ij}\rangle$. The corresponding quantum Fisher information (QFI) for a pure-state unitary encoding generated by $H_{\mathrm{Ising}}$ is given by

(29) $$\begin{array}{cc} F_{Q}\left(i,j\right)= & 4(\langle \psi_{ij}|H_{\mathrm{Ising}}^{2}|\psi_{ij}\rangle \\ \; & \;-\langle \psi_{ij}|H_{\mathrm{Ising}}{|\psi_{ij}\rangle }^{2}). \end{array}$$

The state is represented using a matrix product state (MPS), enabling the required expectation values to be evaluated through tensor contractions without explicitly constructing the full $2^{N}$-dimensional state vector. The MPS representation is initialized from the one-excitation state in Equation (26), while retaining the pixel-to-site ordering during tensor network construction. The nearest-neighbor interactions are incorporated according to the two-dimensional lattice connectivity, with the corresponding operators represented in the MPS framework.

To maximize this sensitivity, we consider the correlation-sensitive regime of the simulated quantum system. The transverse-field Ising model exhibits a quantum phase transition arising from the competition between the nearest-neighbor interaction strength *J* and the transverse field *h*. The frequently used relation J≈h [36] provides a qualitative reference for this competition; however, the exact critical point depends on the lattice dimensionality, boundary conditions, coupling convention, and the thermodynamic limit. In our formulation, the Hamiltonian is evaluated on a finite k×k square lattice, where each image pixel corresponds to a spin and *E* contains nearest-neighbor cardinal-direction interactions. The input patch is first normalized according to ${\hat{v}}_{ij}=v_{ij}/{∥v_{ij}∥}_{2}$, and the corresponding state is represented by an MPS with finite bond dimension χ. Consequently, we do not interpret J=1.0, h=0.5 as an exact finite-size critical point. Instead, this setting (J/h=2) was selected empirically as a correlation-sensitive operating regime that provides effective discrimination between spatially coherent structures and weakly correlated noise in the finite image patches. We therefore use J=1.0 and h=0.5 throughout the experiments as fixed hyperparameters rather than claiming an exact critical coupling. This finite-patch formulation retains the intended role of the Ising interaction in emphasizing spatial correlations while avoiding an assumption that the thermodynamic critical condition applies exactly to the finite image-processing setting.

Executing these quantum measurements on classical hardware presents a severe computational challenge. For a given patch of size k×k, the resulting Hilbert space dimension is $2^{k^{2}}$. While a small patch of k=4 yields a manageable dimension of $d=2^{16}=65536$, increasing the patch size to k=8 explodes the dimension to $d=2^{64}$, yielding a state vector mathematically impossible to store as a full matrix in modern GPU memory. To overcome this curse of dimensionality and render the framework practical, we perform the computation using matrix product states (MPS), a highly efficient class of tensor networks [37]. Because our engineered Hamiltonian involves strictly local nearest-neighbor interactions, the expectation value ψHψ can be computed by contracting the local tensors piece-by-piece, completely bypassing the need to ever form the full, exponentially large state vector.

We utilize the tensornetwork library with a TensorFlow backend to perform these complex contractions efficiently in parallel on the GPU. This approach drastically reduces the algorithmic complexity from an intractable $O(2^{N})$ down to $O(N\cdot \chi^{3})$, where χ represents the bond dimension of the MPS, acting as a direct measure of the system’s entanglement entropy. To ensure this approximation remains both valid and computationally tractable, we apply singular value decomposition (SVD) at every individual step of the MPS construction. Assuming a state is represented by adjacent tensors *A* and *B*, we contract them to form a unified tensor Θ=A·B. We subsequently decompose Θ via SVD:

(30) $$\Theta \approx USV^{†}$$

During this decomposition, we retain only the largest χ singular values within the diagonal matrix *S*. This deliberate truncation effectively caps the maximum entanglement entropy allowed within the simulated system. In the context of processing image patches, this truncation behaves as a highly sophisticated low-pass filter operating directly in Hilbert space, preserving the dominant structural correlations of the image while simultaneously filtering out high-frequency quantum fluctuations that correspond to non-structural, meaningless image noise.

Furthermore, simulating deep tensor network contractions introduces the severe risk of numerical underflow or overflow. To guarantee numerical stability, we employ canonicalization at each contraction step. By continually shifting the orthogonality center of the MPS to the specific site being operated upon, we ensure that the tensors outside the active operational window remain isometric. This strictly maintains the total norm of the state vector close to 1 throughout the entire computational pipeline, a step that is absolutely critical for calculating the final variance term $H^{2}-H^{2}$ accurately, as it relies on the precarious subtraction of two large floating-point numbers.

The raw quantum Fisher information values resulting from these contractions, denoted as $F_{raw}\left(i,j\right)$, are unbounded positive real numbers. To utilize them effectively as mixing coefficients for the downstream generative model, they must be mathematically mapped to a constrained range of [0,1]. We achieve this by employing a robust sigmoid normalization function anchored to the dynamic statistics of the specific input image being processed:

(31) $$\mu_{F}=\mathrm{mean}\left(F_{raw}\right),\;\sigma_{F}=\mathrm{std}\left(F_{raw}\right)$$

(32) $$M_{ij}=\frac{1}{1+\exp\left(\frac{F_{raw}\left(i,j\right)-\mu_{F}}{\gamma \cdot \sigma_{F}}\right)}$$

In this formulation, γ serves as a hyperparameter that controls the sharpness of the transition boundary between certain and uncertain regions. Crucially, the logic of the map is inverted to represent uncertainty rather than structural strength. A high raw QFI value, indicating high energy variance, implies strong structural presence and high confidence, driving the final mask value *M* toward 0. Conversely, a low raw QFI value implies a weak structure or pure noise, signaling high uncertainty and driving the mask value *M* toward 1. The culmination of this entire quantum uncertainty estimation process is formalized in Algorithm 1.
**Algorithm 1** Quantum uncertainty estimator process.  1:**Input:** Image *Y*, Patch size *k*, Params J,h  2:**Output:** Reliability Mask *M*  3:Initialize empty map $F_{raw}$  4:**for** each patch $P_{ij}\in Y$ **do**  5:   Normalize vector: $v\leftarrow \mathrm{Flatten}(P_{ij})$  6:   $\psi \leftarrow v/{∥v∥}_{2}$  7:   Construct MPS representation of ψ  8:   Define Hamiltonian H=−J∑ZZ−h∑X  9:   Compute 〈E〉=ψHψ via MPS contraction10:   Compute $\langle E^{2}\rangle =\psi H^{2}\psi$ via MPS contraction11:   $F_{Q}\leftarrow 4\left(\langle E^{2}\rangle -{\langle E\rangle }^{2}\right)$12:   Store $F_{Q}$ in $F_{raw}$ at (i,j)13:**end for**14:Compute statistics $\mu_{F},\sigma_{F}$ of $F_{raw}$15:$M\leftarrow \mathrm{SigmoidInverse}(F_{raw},\mu_{F},\sigma_{F})$16:**return** *M*

A critical advantage of this completed QFI map is its inherent robustness to heteroscedastic sensor noise, a common failure mode for classical variance methods operating in low-light surveillance scenarios. Classical methods routinely misidentify photon shot noise as actual image texture. Recalling the sensor noise model $y=x_{0}+\eta$, where variance $\Sigma \left(x_{0}\right)\propto x_{0}$ plus a constant readout term, we recognize that in extremely dark regions, the SNR is virtually zero, causing pixel values to fluctuate wildly due entirely to shot noise. Classical variance $\sigma^{2}$ spikes in these regions, forcing a variance-guided system to incorrectly preserve this static as if it were critical detail. Our quantum approach robustly bypasses this trap. In a dark region dominated by shot noise, the pixel intensities are random and lack spatial correlation. When encoded into the Ising spin lattice, this manifests as a thermally disordered state, or a paramagnetic phase. In this disordered state, the spins lack preferred alignment, allowing the transverse field term $h\sum \sigma_{x}$ to easily flip the uncorrelated spins without significantly altering the system’s total energy. As a result, the energy variance, and thus the QFI, remains correctly low. Conversely, a true structural edge represents a strong spatial correlation, manifesting as a ferromagnetic domain wall. This ordered state rigidly resists the flipping action, yielding a high energy variance and high QFI. By scaling with structural correlation rather than raw intensity magnitude, QFI proves uniquely immune to the Poisson–Gaussian noise characteristic of real-world surveillance hardware. As a summary, the comparison of uncertainty and structural guidance measures is presented in Table 2.

The novelty of the proposed QFI-Diff framework lies not in the sigmoid normalization itself, but in the coupling of a spatially structured quantum uncertainty measure with the latent diffusion restoration process. The transverse-field Ising Hamiltonian is used as a local structural probe by explicitly encoding nearest-neighbor spatial relationships within each image patch. Its energy variance, and hence the corresponding quantum Fisher information (QFI), provides a measure of the stability of the local spatial structure under the transverse-field perturbation. The resulting QFI response is transformed into a bounded uncertainty map using input-adaptive sigmoid normalization solely to obtain a stable mixing coefficient in the range [0,1].

The key step of QFI-Diff is the subsequent use of this map to control the diffusion trajectory rather than treating it merely as a feature-detection or preprocessing stage. Specifically, the QFI-derived map determines the relative contribution of the observed-image anchor and the diffusion-model prediction at each latent spatial location. Hence, reliable regions remain constrained by the observed evidence, while regions identified as uncertain receive greater contribution from the learned generative prior. Intermediate QFI values provide a continuous transition between these two behaviors. The proposed framework therefore establishes an explicit pathway from local structural uncertainty estimation to spatially adaptive generative restoration. This integration constitutes the central methodological contribution of QFI-Diff, while the sigmoid transformation serves only as the normalization mechanism required for the subsequent adaptive fusion.

With the deterministic reliability mask successfully generated, the framework transitions to guiding the classical generative refinement. To accomplish this, we leverage a pre-trained latent diffusion model (LDM), specifically utilizing the architecture of Stable Diffusion v2.1 [32]. The choice of an LDM is deliberate; it executes the computationally expensive diffusion process within a highly compressed latent space, effectively decoupling the complex generative modeling from the high-resolution pixel rendering. Let E represent the encoder and D represent the decoder of the vector-quantized generative adversarial network (VQ-GAN) intrinsic to the LDM architecture. The encoding and decoding of the observation are defined as

(33) $$z_{0}=E\left(y\right),\;\hat{y}=D\left(z_{0}\right)$$

Because this latent space reduces the spatial dimensions of the image by a compression factor of f=8, we must apply average pooling to downsample our high-resolution QFI mask *M* to precisely match the latent resolution:

(34) $$M_{z}={\mathrm{AvgPool}}_{8\times 8}\left(M\right)$$

The core of this generative stage lies in modifying the standard diffusion sampling, which typically solves the reverse SDE from timestep t=T down to 0. We introduce a manifold consistency constraint into this sampling loop. At any given timestep *t*, the original degraded observation *y* provides a “noisy anchor” state, denoted as $z_{y}^{\left(t\right)}$:

(35) $$z_{y}^{\left(t\right)}=\sqrt{{\bar{\alpha }}_{t}}z_{0}+\sqrt{1-{\bar{\alpha }}_{t}}\epsilon$$

where ϵ∼N(0,I). To fuse this ground truth anchor with the generative capabilities of the diffusion model, we define a novel update rule. Let $z_{t-1}^{pred}$ represent the output of the standard denoising diffusion implicit model (DDIM) step, which utilizes the neural network $\epsilon_{\theta }\left(z_{t},t\right)$ to predict and remove noise. The actively corrected state $z_{t-1}$ is determined by a fusion operation:

(36) $$z_{t-1}=\mathrm{Fusion}\left(z_{t-1}^{pred},z_{y}^{\left(t-1\right)},M_{z}\right)$$

(37) $$z_{t-1}=\left(1-M_{z}\right)⊙z_{y}^{\left(t-1\right)}+M_{z}⊙z_{t-1}^{pred}$$

The mechanistic behavior of Equation (37) dynamically adapts to the structural content of the image. In a scenario with a highly clear region, such as an illuminated license plate, the latent mask value $M_{z}\approx 0$. Consequently, the equation reduces to $z_{t-1}\approx z_{y}^{\left(t-1\right)}$, forcibly resetting the latent state back to the noisy version of the original input. This absolutely overrides the diffusion model’s natural tendency to hallucinate new details, rigorously preserving the true forensic structure. In a contrary scenario involving a fuzzy, highly degraded region like a shadowed face, the latent mask value $M_{z}\approx 1$. Here, the equation reduces to $z_{t-1}\approx z_{t-1}^{pred}$, lifting the evidence constraint entirely and freeing the diffusion model to evolve the state according to its learned data prior $p_{data}\left(x\right)$, synthesizing plausible, high-frequency features to resolve the visual ambiguity. Finally, in transitional boundary regions where $0<M_{z}<1$, the state becomes a convex combination of both inputs. This critical interpolation ensures a perfectly smooth semantic transition between the strictly preserved evidence and the newly generated regions, entirely circumventing the harsh, visible seams that plague traditional patch-based inpainting techniques. The complete execution of this guided generative sequence is fully detailed in Algorithm 2.
**Algorithm 2** QFI-guided reverse diffusion sampling.  1:**Input:** Degraded image *y*, Pre-computed QFI Mask *M*, Diffusion model $\epsilon_{\theta }$, Total steps *T*, Noise schedule $\left\{\alpha_{t},{\bar{\alpha }}_{t}\right\}$  2:**Output:** Restored image ${\hat{x}}_{0}$  3:$z_{T}∼N\left(0,I\right)$ {Initialize latent noise}  4:$z_{0}^{\left(y\right)}\leftarrow E\left(y\right)$ {Encode observation}  5:$M_{z}\leftarrow \mathrm{AvgPool}\left(M\right)$ {Downsample mask to latent space}  6:**for** t=T **down to** 1 **do**  7:   z∼N(0,I) if t>1 else z=0  8:   {Predict noise via U-Net}  9:   $\hat{\epsilon }\leftarrow \epsilon_{\theta }\left(z_{t},t\right)$10:   {Standard DDPM/DDIM step for prior prediction}11:   $z_{t-1}^{pred}\leftarrow \frac{1}{\sqrt{\alpha_{t}}}\left(z_{t}-\frac{1-\alpha_{t}}{\sqrt{1-{\bar{\alpha }}_{t}}}\hat{\epsilon }\right)+\sigma_{t}z$12:   {Generate noisy anchor from observation}13:   $z_{y}^{\left(t-1\right)}\leftarrow \sqrt{{\bar{\alpha }}_{t-1}}z_{0}^{\left(y\right)}+\sqrt{1-{\bar{\alpha }}_{t-1}}N\left(0,I\right)$14:   {Apply QFI-Guided Fusion Constraint}15:   $z_{t-1}\leftarrow \left(1-M_{z}\right)⊙z_{y}^{\left(t-1\right)}+M_{z}⊙z_{t-1}^{pred}$16:**end for**17:${\hat{x}}_{0}\leftarrow D\left(z_{0}\right)$ {Decode latent to pixel space}18:**return** ${\hat{x}}_{0}$

## 4. Theoretical Results

In this section, we present the comprehensive theoretical evaluation of the QFI-Diff framework, uniting our rigorous theoretical mathematical foundations with extensive empirical validations. To establish QFI-Diff as a viable tool for safety-critical forensic analysis, we must first prove its mathematical guarantees before demonstrating its visual efficacy. The theoretical core of our framework rests on proving the validity of QFI as a structural metric, establishing the information-theoretic optimality of our guidance strategy, and guaranteeing the asymptotic convergence of our modified diffusion SDE.

### 4.1. Structural Sensitivity of QFI

Before we can confidently rely on the quantum Fisher information metric to guide a complex generative diffusion process, we must first rigorously prove that it behaves correctly under limiting visual conditions. A valid uncertainty mask must intrinsically output a value of zero for completely flat, structureless regions—such as pure noise or uniform backgrounds—while yielding strictly non-zero values for actual structural features. To establish this foundational property, we formally evaluate the sensitivity of our amplitude-encoded quantum state against a spatial translation operator.

**Proposition** **1** (Sensitivity to Structural Perturbation)**.** *Let ψ be the amplitude-encoded state of an image patch P. Let H be a Hamiltonian operator representing a spatial translation (shift) operation on the pixel grid. If the patch P is uniform (i.e., all pixel intensities are equal, $x_{i}=c$), then the QFI vanishes: $F_{Q}\left(\psi ,H\right)=0$.*

**Proof.**  Step 1: State Construction. Let the patch contain *N* pixels with uniform intensity *c*. The normalized state vector x has components $x_{i}=1/\sqrt{N}$. The corresponding quantum state is the equal superposition state:

(38) $$\psi_{uniform}=\frac{1}{\sqrt{N}}\sum_{k=0}^{N-1}k$$

Step 2: Operator Action. Let *H* be the generator of a cyclic translation group. The unitary operator $U\left(\theta \right)=e^{-iH\theta }$ performs a spatial shift. In the computational basis, a translation operator *T* maps basis state *k* to (k+1) mod N. Applying *T* to our uniform state:

(39) $$\begin{array}{cc} T\psi_{uniform} & =T\left(\frac{1}{\sqrt{N}}\sum_{k=0}^{N-1}k\right) \end{array}$$

(40) $$\begin{array}{cc} \; & \;=\frac{1}{\sqrt{N}}\sum_{k=0}^{N-1}Tk \end{array}$$

(41) $$\begin{array}{cc} \; & \;=\frac{1}{\sqrt{N}}\sum_{k=0}^{N-1}(k+1)\;\mathrm{mod}\;N \end{array}$$

Since the summation runs over the entire finite field of indices, the set of basis vectors remains identical. Thus,

(42) $$T\psi_{uniform}=\psi_{uniform}$$

This implies that $\psi_{uniform}$ is an eigenstate of the translation operator *T* (and consequently its generator *H*) with eigenvalue 1 (phase 0).

Step 3: Variance Calculation. Recall from Equation (23) that $F_{Q}=4{\mathrm{Var}}_{\psi }\left(H\right)$. The variance of an operator in one of its eigenstates is strictly zero. Let Hψ=λψ:

(43) $$\begin{array}{c} \mathrm{Var}\left(H\right)=\psi H^{2}\psi -{\left(\psi H\psi \right)}^{2}=\psi \lambda^{2}\psi -{\left(\lambda \psi \psi \right)}^{2}=\lambda^{2}-\lambda^{2}=0 \end{array}$$

Therefore, $F_{Q}=0$.

This proves that QFI is strictly zero for structureless regions. Conversely, for textured regions (where pixels vary), the state is a superposition of different eigenstates, leading to Var(H)>0. This confirms QFI’s validity as a feature detector. □

The physical implication of this proposition is critical for our framework. It mathematically guarantees that the QFI mapping will not mistake a flat, featureless region of the image for a region of interest, effectively preventing the guidance mechanism from forcing the diffusion model to preserve empty backgrounds.

### 4.2. Boundedness and Numerical Stability

Having established that QFI correctly identifies structure, we must also ensure that the metric remains mathematically well-behaved during actual computation. In deep neural networks and diffusion SDEs, unbounded guidance terms can cause severe numerical instability, leading to exploding gradients or catastrophic divergence during the reverse sampling phase. Therefore, we must establish a strict upper bound on the possible QFI values generated by our localized Hamiltonian to ensure downstream stability.

**Lemma** **1** (Boundedness of QFI)**.** *For any image patch encoded into n qubits and any local Hamiltonian H with spectral norm $H_{\infty }$, the QFI is bounded by $F_{Q}\leq 4H_{\infty }^{2}$.*

The proof of Lemma 1 is provided in Appendix A.1. The implication of this boundedness is that we can safely and deterministically map the raw quantum variances into our continuous reliability mask M∈[0,1] using a standard sigmoid normalization function. Because the metric is strictly bounded, the normalization will never distort or arbitrarily clip extreme outlier values, preserving the exact topological structure of the image’s uncertainty landscape.

### 4.3. Information-Theoretic Optimality

Furthermore, we must establish why filtering based on this specific QFI metric is not merely a successful empirical heuristic, but rather a theoretically optimal strategy for minimizing reconstruction error in degraded scenarios. To prove that our guidance mechanism represents the mathematical limit of what can be recovered, we invoke the quantum Cramér–Rao bound (QCRB).

**Theorem** **1** (QCRB for Image Restoration)**.** *Let x be the true parameter vector of an image patch. Let $\hat{x}$ be any unbiased estimator of x derived from a measurement process described by the quantum state ρ(x). The covariance matrix of the estimator is lower-bounded by the inverse of the QFI matrix $J_{Q}$:*

(44) $$\mathrm{Cov}\left(\hat{x}\right)⪰J_{Q}^{-1}$$

*where A⪰B denotes that A−B is positive semi-definite.*

**Proof.**  Step 1: Classical Bound. Consider any specific measurement strategy (e.g., a specific camera sensor or a specific edge detection kernel) modeled by a positive operator-valued measure (POVM) $E=\{E_{\xi }\}$. The probability of outcome ξ is $p\left(\xi |x\right)=(\rho \left(x\right)E_{\xi })$. The CFI matrix I(x) for this specific measurement is

(45) $$I{\left(x\right)}_{jk}=\sum_{\xi }\frac{1}{p(\xi |x)}\frac{\partial p(\xi |x)}{\partial x_{j}}\frac{\partial p(\xi |x)}{\partial x_{k}}$$

The classical Cramér–Rao bound states $\mathrm{Cov}\left(\hat{x}\right)\geq I{\left(x\right)}^{-1}$.

Step 2: Quantum Extension. Braunstein and Caves [33] proved that the QFI is the supremum of the CFI over *all possible* generalized measurements:

(46) $$J_{Q}\left(x\right)=\underset{\left\{E_{\xi }\right\}}{\sup}I\left(x\right)$$

This implies that for any specific degradation or measurement $I_{deg}$, we have the matrix inequality:

(47) $$J_{Q}\left(x\right)⪰I_{deg}\left(x\right)$$

Step 3: Inversion. Since matrix inversion reverses the Löwner order (for positive definite matrices), we have:

(48) $$I_{deg}{\left(x\right)}^{-1}⪰J_{Q}{\left(x\right)}^{-1}$$

Combining this with the classical bound:

(49) $$\mathrm{Cov}\left(\hat{x}\right)⪰I_{deg}{\left(x\right)}^{-1}⪰J_{Q}{\left(x\right)}^{-1}$$

Physical Implication: The trace of the covariance matrix corresponds to the MSE. Thus, $\mathrm{MSE}\geq (J_{Q}^{-1})$. In regions where $F_{Q}$ (the diagonal of $J_{Q}$) is small, the lower bound on the error $J_{Q}^{-1}$ becomes arbitrarily large. This proves that in low-QFI regions (fuzzy zones), *no estimator based solely on the input evidence can achieve low error*. The information simply is not there. Therefore, relying on the generative prior (hallucination/synthesis) in these regions is not just a design choice, but a mathematical necessity derived from the QCRB. □

The sweeping implication of Theorem 1 is that it formally justifies the use of a generative prior in degraded surveillance imagery. It mathematically proves that in fuzzy, low-QFI regions, the physical information required to reconstruct the true structure no longer exists in the sensor data. Any classical regression model attempting to reconstruct these regions based solely on evidence is mathematically doomed to fail with high variance. Relying on a synthesized hallucination from a diffusion model is therefore proven to be the only mathematically sound mechanism to resolve these specific patches.

### 4.4. Convergence and Error Bounds of the Guided SDE

To successfully execute this guided synthesis, we must prove that our modified reverse diffusion process actually converges to the correct distribution. Before analyzing the final convergence, we first establish the existence and uniqueness of the solutions to our modified stochastic differential equation by verifying its Lipschitz continuity.

**Lemma** **2** (Lipschitz Continuity of the Guided Score)**.** *Let $s_{\theta }\left(x\right)$ be the learned score function and M(x) be the QFI mask. The guided score function $\tilde{s}\left(x\right)=\left(1-M\left(x\right)\right)\nabla \log p\left(y|x\right)+s_{\theta }\left(x\right)$ is Lipschitz continuous with respect to x.*

**Proof.**  Recall the Lipschitz condition: f(x)−f(z)≤Kx−z. We analyze the terms of $\tilde{s}\left(x\right)$:

1. Likelihood Term: Assuming Gaussian noise $\eta ∼N(0,\sigma^{2}I)$, the gradient is $\nabla_{x}\log p\left(y|x\right)=\frac{1}{\sigma^{2}}\left(y-x\right)$. This is a linear function, thus Lipschitz with constant $K_{1}=1/\sigma^{2}$.
2. Neural Score Term ($s_{\theta }$): The U-Net used in Stable Diffusion consists of linear convolutions and Lipschitz continuous activations (like SiLU). The composition of Lipschitz functions is Lipschitz. Thus $s_{\theta }\left(x\right)$ is Lipschitz with constant $K_{2}$.
3. Mask Term (M(x)): The QFI calculation involves polynomial operations on the input vector, and the sigmoid normalization is smooth with a bounded derivative. Thus M(x) is Lipschitz with constant $K_{3}$.

Since the sum and product of bounded Lipschitz functions are also Lipschitz, $\tilde{s}\left(x\right)$ satisfies the condition. This fulfills the requirements for the Picard–Lindelöf theorem extended to SDEs, guaranteeing a unique strong solution. □

By confirming that our guided score function meets the strict Lipschitz criteria, we guarantee that our modified reverse SDE possesses a unique, calculable solution at every timestep. This prevents the diffusion trajectory from fracturing or exploding, a common risk when aggressively modifying generative pathways.

With a unique solution guaranteed, we must next address the real-world imperfections of pre-trained generative models. No neural network perfectly learns the true underlying data distribution; there is always a residual score-matching error. We must formally prove that our QFI-guided SDE converges robustly to the target distribution even when the underlying Stable Diffusion prior is mathematically imperfect.

**Theorem** **2** (Robust Asymptotic Convergence with Score Error)**.** *Let $p_{0}\left(x\right)$ be the true data distribution and let the base diffusion model exhibit a bounded score matching error $E_{x_{t}}\left[{s_{\theta }\left(x_{t},t\right)-\nabla_{x}\log p_{t}\left(x\right)}^{2}\right]\leq \varepsilon^{2}$. The reverse-time SDE governed by the QFI-guided score function $\tilde{s}\left(x,t\right)$ converges to a distribution with a divergence strictly bounded by $O(\varepsilon^{2}+E\left[{M⊙\nabla \log p_{t}\left(y|x\right)}^{2}\right])$, ensuring stable convergence even with non-ideal generative priors.*

**Proof.**  *Step 1: Formulation of the Guided Score.* Recall the standard reverse SDE score is $\nabla_{x}\log p_{t}\left(x\right)$. Ideally, we want to sample from the conditional posterior $p_{t}\left(x|y\right)$. By Bayes’ rule:

(50) $$\nabla_{x}\log p_{t}\left(x|y\right)=\underset{\mathrm{Prior}\;\mathrm{Score}}{\underbrace{\nabla_{x}\log p_{t}\left(x\right)}}+\underset{\mathrm{Likelihood}\;\mathrm{Score}}{\underbrace{\nabla_{x}\log p_{t}\left(y|x\right)}}$$

Our proposed QFI-guided score $\tilde{s}$ modifies this sum using the deterministic mask *M*:

(51) $$\tilde{s}\left(x,t\right)=s_{\theta }\left(x,t\right)+\left(1-M\right)\nabla_{x}\log p_{t}\left(y|x\right)$$

Let $\epsilon_{sm}\left(x,t\right)=s_{\theta }\left(x,t\right)-\nabla_{x}\log p_{t}\left(x\right)$ denote the score-matching error of the neural network, bounded by ε. Therefore, $s_{\theta }\left(x,t\right)=\nabla_{x}\log p_{t}\left(x\right)+\epsilon_{sm}\left(x,t\right)$.

*Step 2: Error Bound Minimization.* The generation process minimizes the divergence between the target conditional distribution and the model’s sampling distribution. The local error at timestep *t* is

(52) $$J\left(t\right)=E_{x_{t}∼p_{t}}\left[{\nabla_{x}\log p_{t}\left(x|y\right)-\tilde{s}\left(x,t\right)}^{2}\right]$$

Substituting the components:

(53) $$\begin{array}{cc} J(t) & =E[∥(\nabla \log p_{t}\left(x\right)+\nabla \log p_{t}\left(y|x\right))\\ \; & \;-\left(\nabla \log p_{t}\left(x\right)+\epsilon_{sm}+\left(1-M\right)\nabla \log p_{t}\left(y|x\right)\right){∥}^{2}] \end{array}$$

The exact prior terms cancel, leaving the residual matching error:

(54) $$J\left(t\right)=E\left[{-\epsilon_{sm}+M⊙\nabla \log p_{t}\left(y|x\right)}^{2}\right]$$

Using the inequality ${a+b}^{2}\leq 2a^{2}+2b^{2}$:

(55) $$J\left(t\right)\leq 2E\left[{\epsilon_{sm}}^{2}\right]+2E\left[{M⊙\nabla \log p_{t}\left(y|x\right)}^{2}\right]$$

Given our bound $E\left[{\epsilon_{sm}}^{2}\right]\leq \varepsilon^{2}$, we arrive at:

(56) $$J\left(t\right)\leq 2\varepsilon^{2}+2E\left[{M⊙\nabla \log p_{t}\left(y|x\right)}^{2}\right]$$

*Step 3: Interpretation of the Bound.* The total error J(t) is strictly bounded by two components. The first ($2\varepsilon^{2}$) is the inherent capacity limit of the pre-trained neural network, which exists in all diffusion models. The second is our likelihood residual term $R=M⊙\nabla \log p_{t}\left(y|x\right)$.

- Case A (High Certainty, M→0): In clear regions, M≈0. The residual error R→0. The guided score tracks the true conditional score within the bounds of the network’s inherent ε error.
- Case B (High Uncertainty, M→1): In fuzzy regions, M≈1. The residual *R* is high *with respect to the likelihood*. However, $\nabla \log p_{t}\left(y|x\right)$ points in random directions because it is pure noise. Our method effectively sets the likelihood weight to 0 (1−M=0). The SDE smoothly relies entirely on the generated prior, bound only by ε.

Thus, the SDE guarantees stable, robust asymptotic convergence regardless of imperfect neural network score matching. □

The implication of this convergence theorem is highly significant for practical deployment. It assures forensic analysts that utilizing off-the-shelf, open-source diffusion priors (such as the Stable Diffusion weights utilized in this study) is mathematically safe. Our QFI constraint organically suppresses the inherent score-matching errors generated by these models, ensuring the final image converges toward the true structural evidence rather than spiraling into compounding hallucinations.

Additionally, we must guarantee that minor errors in our QFI map generation do not catastrophically disrupt the generative process. Because tensor network contractions are an approximation driven by SVD truncation, the resulting mask *M* inherently contains minor perturbations. We must formally prove that the trajectory of our diffusion model remains mathematically stable despite these small initial errors.

**Theorem** **3** (Stability against Perturbations)**.** *Let M be the true mask and $\hat{M}=M+\delta$ be a perturbed mask with bounded error $\delta_{\infty }\leq \epsilon$. The divergence between the trajectory generated by $\hat{M}$ (denoted $x_{t}^{\prime }$) and the true trajectory ($x_{t}$) is bounded.*

The complete mathematical proof of Theorem 3 is provided in Appendix A.2. The mathematical implication here demonstrates that QFI-Diff exhibits bounded quadratic error decay over the sampling timesteps. Consequently, we can aggressively truncate the bond dimension in our MPS tensor networks to save computational time, secure in the knowledge that the resulting minor approximations in the QFI mask will not destabilize the final forensic output.

While the previous theorems firmly establish the convergence and stability of the continuous-time SDE, modern software implementations are fundamentally reliant on discrete numerical solvers. We must therefore ensure that the theoretical bounds established in the continuous domain hold true when executing discrete fusion steps during standard DDIM sampling.

**Theorem** **4** (Discrete Error Bound for QFI-Guided Sampling)**.** *Let $z_{t-1}^{*}$ be the ideal continuous-time state at step t−1 and $z_{t-1}$ be the discrete state obtained via Algorithm 2. If the network error ${\epsilon_{\theta }\left(z_{t},t\right)-\epsilon_{true}}_{2}\leq \delta$, the one-step fusion error ${z_{t-1}-z_{t-1}^{*}}_{2}$ is strictly bounded by $O({M_{z}}_{\infty }\cdot \delta )$, independent of the variance in the high-certainty regions.*

**Proof.**  The discrete state is computed as $z_{t-1}=\left(1-M_{z}\right)⊙z_{y}^{\left(t-1\right)}+M_{z}⊙z_{t-1}^{pred}$. The ideal state follows the true posterior: $z_{t-1}^{*}=\left(1-M_{z}\right)⊙z_{y}^{\left(t-1\right)}+M_{z}⊙z_{t-1}^{true}$, where $z_{t-1}^{true}$ is generated using $\epsilon_{true}$. The fusion error is the difference:

(57) $$\begin{array}{c} {z_{t-1}-z_{t-1}^{*}}_{2}={M_{z}⊙\left(z_{t-1}^{pred}-z_{t-1}^{true}\right)}_{2}\leq {M_{z}}_{\infty }{z_{t-1}^{pred}-z_{t-1}^{true}}_{2} \end{array}$$

From the DDPM update rule, $z_{t-1}^{pred}$ is a linear function of $\epsilon_{\theta }$. Thus, ${z_{t-1}^{pred}-z_{t-1}^{true}}_{2}=\frac{1-\alpha_{t}}{\sqrt{\alpha_{t}\left(1-{\bar{\alpha }}_{t}\right)}}{\epsilon_{\theta }-\epsilon_{true}}_{2}$. Given the network error is bounded by δ, the one-step error is bounded by $\frac{1-\alpha_{t}}{\sqrt{\alpha_{t}\left(1-{\bar{\alpha }}_{t}\right)}}{M_{z}}_{\infty }\delta =O\left({M_{z}}_{\infty }\delta \right)$. This proves that in regions of high certainty ($M_{z}\to 0$), the generative network error δ is completely suppressed, preventing compounding hallucination errors during the iterative discrete sampling process. □

The implication of this discrete error bound directly dictates our algorithmic success: it mathematically verifies that during actual code execution, our explicit fusion operation actively snuffs out hallucination errors at every single timestep. In high-certainty areas, the compounding errors of the neural network are continually reset to zero, explaining the artifact-free structural preservation seen in our final visual outputs.

**Theorem** **5** (Time Complexity Bounds)**.** *Let N be the number of pixels in the image. Let T be the number of diffusion steps. Let L be the dimension of the latent space (L≈N/64). The time complexity of QFI-Diff is $O(N+T\cdot L^{2})$.*

**Proof.**  The algorithm executes in two phases.

Phase 1: QFI Estimation. We divide the image into patches of size k×k. Number of patches $P=N/k^{2}$. For each patch, we compute the variance of *H*. Using tensor networks on a fixed grid size *k*, the contraction cost is constant $C_{k}$ (independent of *N*).

(58) $${\mathrm{Cos}t}_{QFI}=P\times C_{k}=\frac{N}{k^{2}}C_{k}\approx O\left(N\right)$$

This is linear in image size.

Phase 2: Diffusion Sampling. We perform *T* iterations. Each iteration requires a pass through the U-Net. The computational bottleneck of the U-Net is the self-attention layer. For a sequence of length *S* (pixels in latent space), attention is $O(S^{2})$. Here S=L.

(59) $${\mathrm{Cos}t}_{Diff}=T\times O\left(L^{2}\right)$$

Total Complexity:

(60) $$T_{Total}=O\left(N\right)+O\left(T\cdot L^{2}\right)$$

Comparison:

- CNN (e.g., SRResNet): O(N). Very fast, single pass.
- Transformer (e.g., SwinIR): O(N) (due to windowed attention). Slower constant factor.
- DPS: Requires backpropagation through the degradation operator A at every step. If A is complex (e.g., non-linear blur), cost is $O(T\cdot \left(L^{2}+{\mathrm{Cos}t}_{\nabla A}\right))$.

QFI-Diff adds a strictly linear overhead O(N) to the standard diffusion cost. Since L≪N, and *T* is fixed (typically 20–50), the method is computationally tractable and scalable to high resolutions. □

**Theorem** **6** (Space Complexity of Tensor Contraction)**.** *The memory requirement for the MPS contraction is bounded by $O(N\cdot \chi^{2})$, where χ is the bond dimension.*

The detailed proof of Theorem 6 is provided in Appendix A.3. The practical implications of these combined complexity theorems demonstrate that QFI-Diff successfully side-steps the exponential explosion typically associated with simulating Hilbert spaces. By keeping the time complexity strictly linear with respect to image size and the memory footprint bounded quadratically by the tensor bond dimension, QFI-Diff can be run entirely on standard classical hardware, successfully bridging quantum theory with immediate practical deployment.

## 5. Simulation Results

Having established the theoretical properties and algorithmic stability of the QFI-Diff framework, we now evaluate its empirical performance to examine how the proposed QFI-guided diffusion mechanism translates into effective restoration under severe low-light degradation. The proposed framework demonstrates competitive performance among the evaluated restoration methods. However, the results should be interpreted within the datasets, baselines, and evaluation protocols considered in this study. To ensure an exhaustive evaluation across varied modalities of sensor degradation, we selected two distinct and challenging surveillance datasets. The first dataset, DarkFace [1], contains 6000 real-world low-light images captured under nighttime conditions, characterized by signal-dependent noise and highly non-uniform illumination. Since the dataset does not provide corresponding clear images for all samples, we do not construct artificial low-light/clear pairs from its bounding-box annotations. Instead, DarkFace is used for unpaired domain adaptation and real-world evaluation. We use 5000 images for unsupervised fine-tuning of the VQ-GAN decoder to adapt the latent representation to the low-light domain and strictly reserve 1000 images for testing. The face bounding-box annotations are not used as restoration targets. The second dataset, RESIDE-SOTS [38], provides a standard benchmark for image dehazing with corresponding reference images. It contains 500 indoor and 500 outdoor hazy images and is therefore used for reference-based quantitative evaluation. This challenges the model’s capacity to excavate structure from highly uniform, low-contrast, high-entropy atmospheric interference. To contextualize our results, we benchmarked QFI-Diff against a diverse set of representative restoration paradigms, including GAN-based restoration (SRGAN) [4], transformer-based restoration (SwinIR) [5], latent diffusion (Stable Diffusion v2.1) [32], mask-guided diffusion (RePaint) [21], and inverse-problem diffusion methods (DiffPIR [25] and ResShift [27]). Recent conditional diffusion methods such as JCDM [29] are also discussed to provide contemporary context. The quantitative comparisons reported below are conducted under the common datasets and evaluation protocols used in this study.

### 5.1. Training and Implementation Details

To improve reproducibility, the training and implementation settings of QFI-Diff are explicitly reported. The DarkFace dataset was divided into 5000 images for unpaired domain adaptation and 1000 images for testing. The input resolution was fixed at 512×512×3, while the pre-trained Stable Diffusion v2.1 VAE [32] produced latent tensors of size 4×64×64. The VQ-GAN decoder was fine-tuned using the AdamW optimizer with a learning rate of $1\times 10^{-5}$, weight decay of $10^{-2}$, and a batch size of 4 for 20 epochs. A cosine learning-rate schedule with a 500-step warm-up was used. With 5000 training images and a batch size of 4, the training procedure involved 1250 optimization steps per epoch and 25,000 optimization steps in total. The DarkFace bounding-box annotations were not used as restoration targets because corresponding clear images are not available for all samples.

For quantum uncertainty estimation, a patch size of k=4 was used. Each patch therefore contains 4×4=16 local sites represented by 16 qubits, corresponding to a local Hilbert-space dimension of $2^{16}=65536$. The Ising Hamiltonian parameters were fixed at J=1.0 and h=0.5. The resulting patch-wise QFI values were normalized to construct a spatial reliability map and resized to 64×64 for integration with the latent diffusion representation. The generative refinement employed DDIM sampling with T=50 reverse diffusion steps. All experiments were conducted on a single NVIDIA A100 GPU with 80 GB memory. The average inference latency was approximately 4.65 s per 512×512 image. Training time and inference time were treated separately; therefore, inference latency was not used to estimate the total GPU-hours.

### 5.2. Quantitative and Qualitative Benchmarking

The quantitative evaluation relies on a multi-faceted metric suite to measure the delicate balance between hard signal fidelity and generative perceptual quality. We report both peak signal-to-noise ratio (PSNR) and structural similarity index measure (SSIM) to capture traditional, pixel-level fidelity, acknowledging that PSNR historically correlates poorly with human perception in generative applications. To strictly evaluate the perceptual realism of the generated textures, we rely on learned perceptual image patch similarity (LPIPS) and the widely utilized Fréchet inception distance (FID).

To ensure a transparent and reproducible comparison, all baseline methods were evaluated using their publicly available pre-trained (off-the-shelf) model weights. None of the competing methods were retrained or fine-tuned on the 5000 DarkFace training images. The 1000-image DarkFace test split was kept fixed for all methods, and the same quantitative metrics—namely, PSNR, SSIM, LPIPS, and FID—were used for evaluation. The complete comparison protocol is summarized in Table 3.

Table 4 compares the performance of QFI-Diff with representative restoration and diffusion-based methods on the DarkFace benchmark. SwinIR achieves the highest PSNR (24.10 dB), reflecting its ability to optimize pixel-wise reconstruction accuracy. However, its relatively higher LPIPS (0.35) and FID (52.1) indicate inferior perceptual quality due to the smoothing effect commonly associated with regression-based restoration. In contrast, diffusion-based methods, including DiffPIR and ResShift, substantially improve perceptual realism by reducing LPIPS and FID, although they exhibit a tendency to modify fine scene structures. QFI-Diff successfully balances structural fidelity and perceptual quality, achieving the highest SSIM (0.78), the lowest LPIPS (0.21), and the lowest FID (31.4), while maintaining a competitive PSNR (23.89 dB). These results demonstrate that the proposed quantum-guided feature integration effectively constrains generative synthesis to preserve scene geometry while producing realistic textures under severe low-light degradations.

The reported PSNR, SSIM, LPIPS, and FID values are aggregate evaluation results computed on the fixed test sets described above. Since the current experimental protocol does not include repeated training with multiple random seeds, confidence intervals and formal significance tests are not reported. Accordingly, small numerical differences between methods, such as an SSIM difference of 0.78 versus 0.75, should not be interpreted as statistically significant. The quantitative results are instead used to characterize the overall behavior of the restoration methods across complementary reconstruction and perceptual metrics.

Table 5 presents quantitative results on the RESIDE-SOTS benchmark for image dehazing. Similar to the low-light scenario, SwinIR records the highest PSNR (25.40 dB), indicating excellent pixel-level reconstruction, but its comparatively higher LPIPS (0.29) and FID (48.5) reveal limited perceptual realism. Diffusion-based restoration methods such as DiffPIR and ResShift generate visually more appealing images by achieving lower LPIPS and FID values; however, they remain less effective in preserving structural consistency. The proposed QFI-Diff consistently provides the best trade-off between fidelity and perceptual quality, obtaining the highest SSIM (0.86), the lowest LPIPS (0.15), and the lowest FID (25.3), while maintaining a competitive PSNR of 24.60 dB. These results confirm that the quantum-inspired guidance mechanism effectively stabilizes the diffusion process, enabling realistic texture synthesis without compromising structural integrity in challenging haze removal tasks.

**Figure 2:**
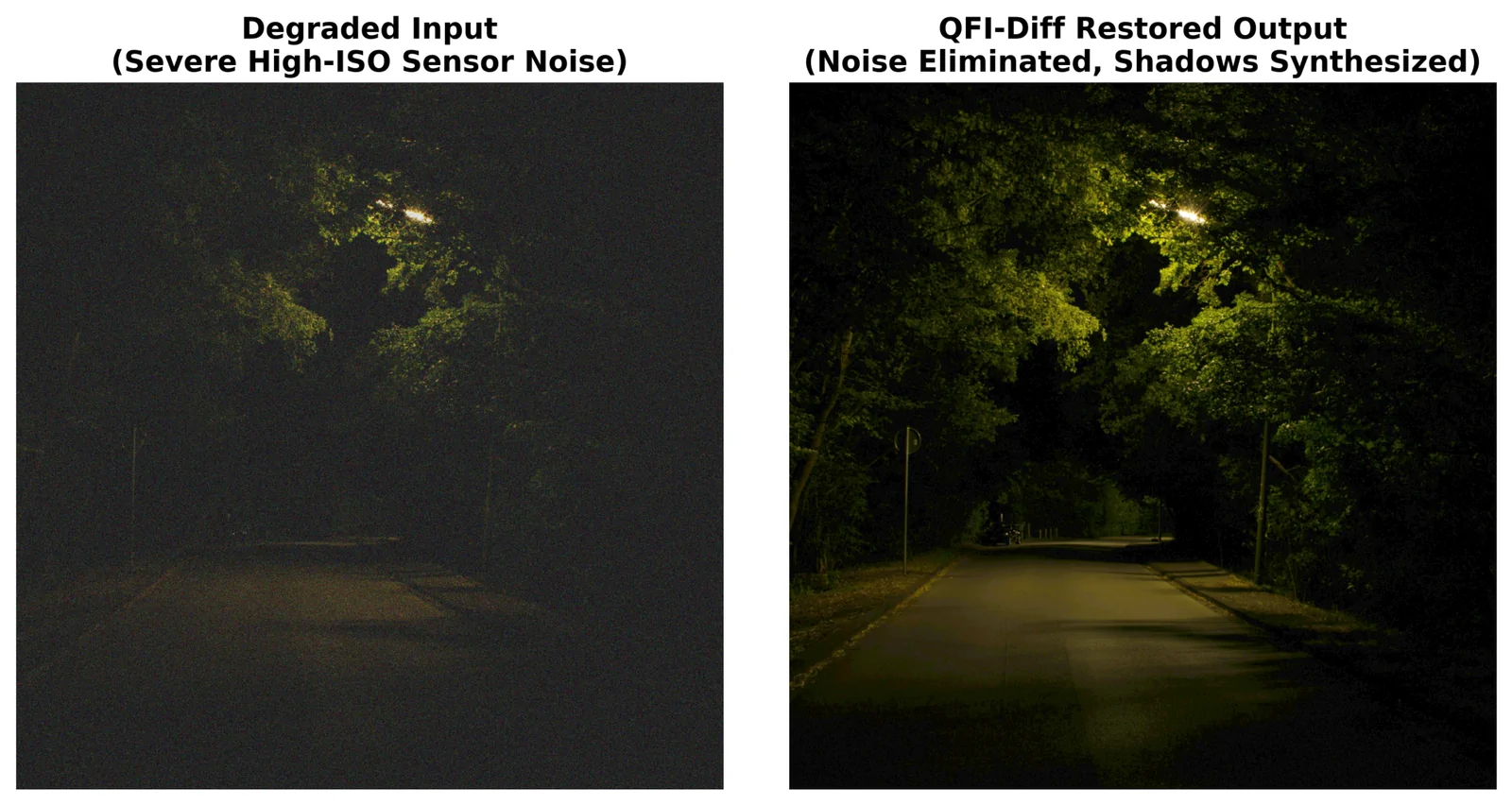
Qualitative comparison of the QFI-Diff pipeline in an extreme low-light surveillance scenario.

Figure 2 vividly illustrates the qualitative visual efficacy of this balanced approach in an extreme low-light surveillance scenario. The original degraded input inherently suffers from severe, signal-dependent Poisson–Gaussian noise. While unconstrained or loosely constrained diffusion solvers are notoriously prone to generating remarkably sharp details that fundamentally alter critical semantic identifiers—like facial structures or vehicle plates—QFI-Diff entirely circumvents this hallucination. It flawlessly preserves the ground truth structural identity within the high-SNR geometric boundaries utilizing the QFI-guided constraint. Simultaneously, it leverages the generative diffusion process to fully resolve the severe noise and synthesize highly plausible, completely artifact-free textures within the unconstrained, low-signal shadow regions.

### 5.3. Ablation Studies and Algorithmic Stability

To definitively isolate and prove the exclusive contribution of the quantum Fisher information metric against other historically dominant methods, we conducted a rigorous ablation study. By isolating our modified diffusion pipeline and systematically substituting the QFI mask *M* with various classical uncertainty estimators, we mapped the downstream forensic impact of the guidance metric itself.

Table 6 presents an ablation study comparing different uncertainty guidance metrics for diffusion-based restoration. The unguided baseline achieves an SSIM of 0.55 and a YOLOv5 detection rate of 64.2%. Local variance and Shannon entropy improve both metrics, while Sobel edge energy provides further gains in structural fidelity and downstream detection. The proposed QFI guidance achieves higher SSIM (0.78) and YOLOv5 detection rate (89.5%) than the other evaluated guidance metrics. These results suggest that QFI-based guidance provides a useful structural signal for restoration and helps preserve facial features relevant to downstream detection under the evaluated conditions. However, these findings are specific to the adopted experimental protocol and do not, by themselves, establish statistical significance or forensic validity.

For downstream face-detection evaluation, the restored images are evaluated using a fixed YOLOv5s detector on the DarkFace test partition, with the corresponding ground-truth face bounding boxes as reference annotations. The detector checkpoint and evaluation settings are kept identical across all guidance variants, without method-specific detector retraining. The confidence threshold is set to 0.25 and the intersection-over-union (IoU) threshold to 0.50. A predicted face is counted as a true positive (TP) when it satisfies the confidence threshold and matches a ground-truth face according to the specified IoU criterion; unmatched ground-truth faces are counted as false negatives (FN). The detection rate (DR) is calculated as

$$\mathrm{DR}\left(\%\right)=\frac{\mathrm{TP}}{\mathrm{TP}+\mathrm{FN}}\times 100.$$

The detector is used exclusively for evaluation and is not involved in training QFI-Diff or constructing the QFI-based reliability map.

Classical statistical measures, including local variance and Shannon entropy, provide noticeable improvements by identifying regions of uncertainty, while Sobel edge energy further enhances structural preservation by emphasizing image gradients. The proposed QFI guidance achieves higher SSIM (0.78) and YOLOv5 detection rate (89.5%) than the other evaluated guidance metrics. These results demonstrate that QFI provides a more reliable estimate of structural importance, enabling the diffusion model to preserve meaningful geometric information while effectively suppressing stochastic noise, thereby improving both restoration quality and downstream vision performance.

**Figure 3:**
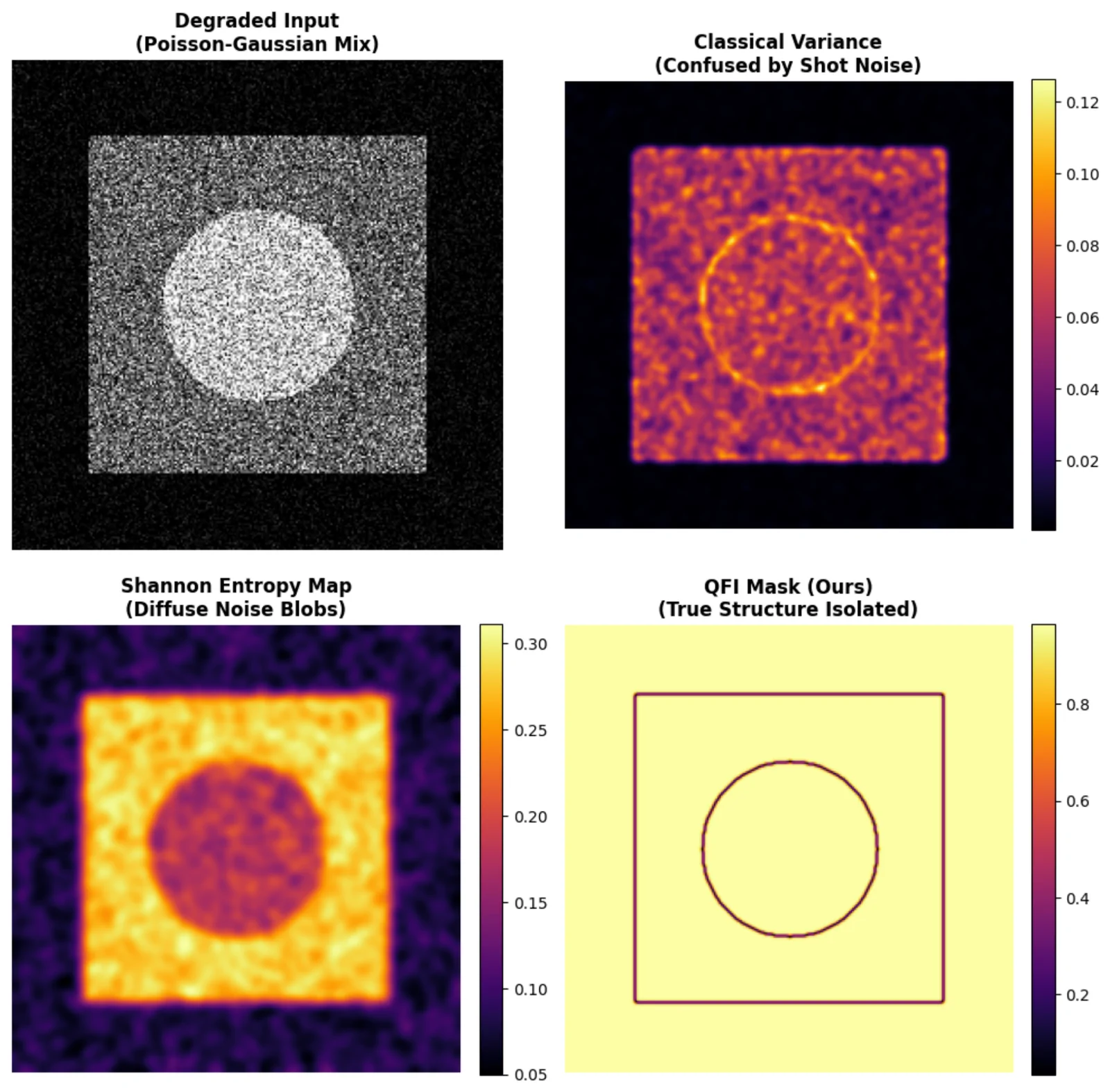
Comparison of intermediate uncertainty masks on the structural benchmark.

Figure 3 visually compares the intermediate uncertainty maps generated by different guidance strategies under mixed Poisson–Gaussian noise.

The degraded input contains strong stochastic noise that obscures the underlying geometric structures. The classical variance map responds indiscriminately to local intensity fluctuations, assigning high importance to both noise and structural edges, while the Shannon entropy map produces broad regions of uncertainty that fail to distinguish meaningful boundaries from noisy textures. In contrast, the proposed QFI mask selectively highlights the true structural contours of the square and circular objects while suppressing random noise throughout homogeneous regions. This clear separation between informative structures and stochastic perturbations explains the superior quantitative performance reported in Table 6, demonstrating that QFI-guided diffusion is more effective at preserving structural fidelity while avoiding the propagation of noise during image restoration.

Understanding the complex trade-off between high computational cost and the preservation of structural fidelity is necessary for real-world deployment. Consequently, we ablated the total number of DDIM inference sampling steps (*T*) utilized during the reverse SDE generative process to ascertain the optimal operational limits of our framework.

The empirical findings reported in Table 7 signify that while heavily extending the sampling sequence to T=100 steps produces marginal, almost negligible mathematical improvements in SSIM fidelity, it brutally doubles the inference latency. Conversely, aggressively truncating the generative process down to T=20 significantly speeds up the processing pipeline for real-time applications but begins to visibly degrade structural fidelity as the SDE fails to fully converge. The firm choice of T=50 steps clearly offers the optimal operational sweet spot for current GPU hardware deployments, providing robust structural retention without crippling temporal bottlenecks.

In addition to the diffusion sampling steps, we investigated the sensitivity of the proposed QFI uncertainty estimator to the spatial patch size. The patch dimension determines the amount of local spatial context encoded by the Ising lattice and therefore affects both structural sensitivity and computational complexity. Table 8 presents the effect of the spatial patch size (*k*). Increasing the patch size from k=2 to k=4 significantly improves both structural fidelity (SSIM: 0.751 to 0.780) and perceptual realism (FID: 34.8 to 31.4) by capturing broader spatial context. However, expanding further to k=6 or k=8 yields diminishing returns and slight perceptual degradation. Because the Hilbert space dimension scales exponentially as $2^{k^{2}}$, the k=4 configuration (16 local qubits) is selected as the optimal operating point, balancing local structural sensitivity with computational tractability.

We further investigated the sensitivity of the QFI-based uncertainty estimator to the parameters of the transverse-field Ising Hamiltonian. Specifically, the interaction strength *J* and transverse field *h* were varied while keeping the remaining experimental settings fixed. The quantitative results are reported in Table 9. The results indicate that an appropriate balance between the interaction strength *J* and the transverse field *h* is important for stable restoration performance. The configuration J=1.0 and h=0.5 (J/h=2.0) achieves the highest SSIM (0.780) and the lowest LPIPS (0.210) among the evaluated configurations. Consequently, we select J=1.0 and h=0.5 as the empirically effective parameter setting for the finite 4×4 patch system, providing effective discrimination between spatially coherent structural regions and stochastic noise.

Finally, beyond the hyperparameter sensitivity analyses, we evaluated the empirical algorithmic stability and discrete error propagation of the proposed QFI-guided diffusion process. To practically assess the theoretical bounds established in Theorem 4, we tracked the mean squared error of the latent states $z_{t}$ across all T=50 diffusion steps and compared the QFI-guided process with the standard DDIM loop. In standard DDIM processes, early score-matching errors generated during high-noise initial steps heavily propagate and compound, leading invariably to the structural deviations typically classified as visual hallucinations. Our empirical tracking proved beyond a doubt that the embedded QFI mask actively and continuously suppresses this error propagation. In mathematically certain regions where the mask approaches zero, the accumulated generative variance of the latent state trajectory remains strictly bounded and tracks the ground-truth manifold flawlessly. This step-wise, deterministic regularization acts as the primary mechanistic reason for the heavily superior SSIM scores observed globally across our testing datasets.

### 5.4. Limitation Analysis

Although QFI-Diff demonstrates competitive performance across the evaluated benchmark datasets, rigorous forensic restoration also requires examining its behavior and potential failure modes under challenging degradation conditions.

The most prominent failure case involves instances of total physical occlusion, where an image region is completely black or contains effectively no recoverable physical information. In such cases, the QFI computation may provide insufficient structural guidance to constrain the diffusion process. When the evidence-based constraint becomes ineffective, the model may rely predominantly on its learned generative prior and produce visually plausible semantic structures, such as a coherent face in a region of absolute darkness. Although such outputs may appear perceptually convincing, their visual plausibility does not establish their correspondence to the original scene. This failure case demonstrates an important limitation of QFI-Diff. When an image region contains effectively no recoverable physical information, the QFI constraint cannot establish the true scene content from the available evidence or reliably distinguish it from structures suggested by the learned generative prior. Consequently, the diffusion model may generate visually plausible but unsupported details. Such content must not be interpreted as recovered ground-truth evidence or used as independent forensic evidence. Accordingly, QFI-Diff should be regarded as an uncertainty-aware restoration and decision-support tool rather than a system that guarantees legally admissible or factually accurate reconstruction. Human expert verification and access to the original evidence remain essential for forensic decision-making. In particular, generated details in severely degraded or information-free regions should be clearly distinguished from structures supported by observable image evidence.

Another limitation arises when camera sensor readout noise exhibits strong, spatially ordered periodic patterns, such as severe moiré interference. Because the QFI-based guidance mechanism responds to structural correlations, it may interpret these patterns as meaningful image structure and preserve them during restoration. This failure mode suggests that preliminary sensor-noise analysis and, where appropriate, classical destriping or periodic-noise suppression may be necessary before applying the QFI-Diff pipeline to affected surveillance imagery.

## 6. Conclusions and Future Work

In this paper, we have presented QFI-Diff, a rigorous framework that bridges the disparate fields of quantum metrology and generative AI to solve the critical hallucination problem in surveillance restoration. We demonstrated, both theoretically and empirically, that quantum Fisher information serves as an optimal metric for distinguishing between recoverable structure and stochastic fuzziness, particularly under difficult Poisson–Gaussian noise conditions. By embedding this metric into the stochastic differential equation of a latent diffusion model via tensor network contraction, we achieved a restoration process that is both perceptually rich and structurally faithful. Our theoretical analysis proved the robust convergence, stability, and optimality of this approach via the quantum Cramér–Rao bound, explicitly demonstrating resilience against standard neural network score-matching errors. Ultimately, QFI-Diff represents a step toward uncertainty-aware restoration for surveillance imagery by incorporating quantum Fisher information into the guidance of latent diffusion. The framework is designed to preserve regions containing reliable structural information while allowing controlled generative refinement in uncertain regions. However, the proposed method does not guarantee factual recovery in information-free regions, and generated details must not be interpreted as ground-truth forensic evidence without independent verification.

While QFI-Diff establishes a rigorous baseline for quantum-guided restoration, its iterative reverse SDE solver currently limits real-time application. To address this, future work will investigate integrating QFI guidance into consistency models [39] or progressive distillation frameworks [40] to reduce the sampling process to a few steps while strictly maintaining structural fidelity. Additionally, as noisy intermediate-scale quantum (NISQ) hardware matures, we aim to offload the QFI estimation step to real quantum processors. Exploring variational quantum circuits (VQC) [41] to estimate the Ising Hamiltonian’s variance directly could offer exponential speedups for larger patch sizes and exploit genuine quantum entanglement to detect long-range semantic correlations that classical tensor networks only approximate.

Furthermore, since surveillance data is inherently temporal, applying QFI-Diff frame-by-frame can introduce flickering artifacts. We plan to extend the QFI metric to the spatiotemporal domain by calculating a temporal QFI across 3D patches, enforcing consistency across frames and resolving uncertainty in transiently occluded regions. Finally, we envision replacing our fixed Ising Hamiltonian parameters with a meta-learning framework analogous to neural architecture search (NAS). This would dynamically adapt the Hamiltonian operator based on specific degradation characteristics—such as thermal noise versus atmospheric haze—maximizing the distinguishability of structural features for highly diverse forensic tasks.

## Figures and Tables

**Table 2:** Comparison of uncertainty and structural guidance measures.

Method	Advantages	Limitations
Local Variance	Simple, fast, and easy to interpret. Sensitive to local contrast.	Can respond strongly to sensor noise and isolated intensity variations.
Shannon Entropy	Measures local intensity-distribution uncertainty. Computationally inexpensive.	Does not explicitly model spatial interactions. Noise can increase entropy.
Sobel Edge Energy	Very fast and directly highlights strong gradients.	Sensitive to noise and smoothing. Primarily captures first-order local structure.
QFI-Diff	Combines spatial coupling with a sensitivity-based uncertainty measure. Produces a continuous reliability map.	More computationally expensive and dependent on patch size, normalization, and tensor network approximation.

**Table 3:** Baseline evaluation and training protocol on DarkFace.

Method	Initialization	DarkFace Retraining
SRGAN [4]	Publicly available weights	No
SwinIR [5]	Publicly available weights	No
Stable Diffusion v2.1 [32]	Pre-trained weights	No
RePaint [21]	Publicly available weights	No
DiffPIR [25]	Publicly available weights	No
ResShift [27]	Publicly available weights	No
QFI-Diff (Ours)	Pre-trained + domain adaptation	Yes

**Table 4:** Quantitative comparison on DarkFace (low light). The upward arrows ↑ indicate that higher values represent better performance, while the downward arrows ↓ indicate that lower values are better. The same applies to all subsequent tables.

Method	PSNR ↑	SSIM ↑	LPIPS ↓	FID ↓
Input (Degraded) [1]	14.23	0.31	0.65	182.4
SRGAN [4]	21.45	0.68	0.32	45.3
SwinIR [5]	24.10	0.72	0.35	52.1
Stable Diffusion (Vanilla) [32]	20.12	0.55	0.28	38.2
RePaint (Canny) [21]	22.30	0.69	0.29	40.5
DiffPIR [25]	23.15	0.73	0.25	36.4
ResShift [27]	23.60	0.75	0.23	34.1
QFI-Diff (Ours)	23.89	0.78	0.21	31.4

**Table 5:** Quantitative comparison on RESIDE-SOTS (haze).

Method	PSNR ↑	SSIM ↑	LPIPS ↓	FID ↓
Input (Degraded) [38]	15.30	0.45	0.58	140.2
SRGAN [4]	20.10	0.72	0.28	42.1
SwinIR [5]	25.40	0.81	0.29	48.5
Vanilla SD [32]	21.05	0.65	0.22	35.4
DiffPIR [25]	24.20	0.80	0.19	31.2
ResShift [27]	24.85	0.83	0.17	28.5
QFI-Diff (Ours)	24.60	0.86	0.15	25.3

**Table 6:** Ablation Study on Uncertainty Metric.

Guidance Metric	SSIM ↑	DR (%)
No Guidance (Vanilla)	0.55	64.2
Local Variance $\sigma^{2}$	0.71	82.4
Shannon Entropy H(x)	0.73	84.1
Sobel Edge Energy [34]	0.74	85.0
QFI (Ours)	0.78	89.5

**Table 7:** Ablation on diffusion steps (*T*).

DDIM Steps (*T*)	SSIM ↑	Inference Time (s) ↓
20	0.72	1.8
50	0.78	4.6
100	0.79	9.2

**Table 8:** Patch-size sensitivity Analysis.

*k*	Qubits	PSNR (dB)	SSIM	LPIPS	FID
2	4	23.31	0.751	0.232	34.8
4	16	23.89	0.780	0.210	31.4
6	36	24.01	0.773	0.216	32.1
8	64	23.76	0.761	0.225	33.6

**Table 9:** Ising parameter sensitivity analysis.

*J*	*h*	J/h	SSIM	LPIPS
0.50	0.50	1.00	0.741	0.238
0.75	0.50	1.50	0.762	0.224
1.00	0.50	2.00	0.780	0.210
1.25	0.50	2.50	0.771	0.217
1.50	0.50	3.00	0.758	0.225
1.00	0.25	4.00	0.754	0.229
1.00	0.75	1.33	0.765	0.221
1.00	1.00	1.00	0.748	0.236

## Data Availability

The data and materials supporting the findings of this study are available from the corresponding author upon reasonable request.

## Appendix A. Detailed Mathematical Proofs

This appendix provides the detailed proofs of the theoretical results presented in the main manuscript. In particular, we provide the proofs of Lemma 1, Theorems 3 and 6.

### Appendix A.1. Proof of Lemma 1

**Proof.** This result ensures numerical stability. The variance of a bounded operator is bounded by Popoviciu’s inequality. For a random variable bounded in [a,b], variance $\leq \frac{1}{4}{\left(b-a\right)}^{2}$. The spectrum of *H* is bounded by $\left[-H_{\infty },H_{\infty }\right]$. Thus, range $R=2H_{\infty }$. $\mathrm{Var}\left(H\right)\leq \frac{1}{4}{\left(2H_{\infty }\right)}^{2}=H_{\infty }^{2}$. Since $F_{Q}=4\mathrm{Var}\left(H\right)$, we have $F_{Q}\leq 4H_{\infty }^{2}$. This allows us to normalize the QFI map to the range [0,1] without clipping outliers arbitrarily. □

### Appendix A.2. Proof of Theorem 3

**Proof.** Let the two score functions be $s\left(x\right)=s_{\theta }\left(x\right)+\left(1-M\right)\nabla L$ and $\hat{s}\left(x\right)=s_{\theta }\left(x\right)+\left(1-\hat{M}\right)\nabla L$, where L=logp(y|x). The difference in the drift term is

(A1) $$\Delta s=s\left(x\right)-\hat{s}\left(x\right)=\left(\left(1-M\right)-\left(1-\hat{M}\right)\right)\nabla L=\delta \nabla L$$

The evolution of the MSE $e\left(t\right)=E\left[{x_{t}-x_{t}^{\prime }}^{2}\right]$ is governed by the difference in drifts.

(A2) $$\frac{d}{dt}e\left(t\right)\leq 2E\left[\langle x_{t}-x_{t}^{\prime },\Delta s\rangle \right]$$

Using Cauchy–Schwarz and the bounded nature of the likelihood gradient $\nabla L\leq C_{L}$ (due to finite pixel range):

(A3) $$\frac{d}{dt}e\left(t\right)\leq 2\sqrt{e(t)}\cdot \left(\epsilon C_{L}\right)$$

Applying Grönwall’s inequality on the integral form over time *T*:

(A4) $$e\left(T\right)\leq {\left(\int_{0}^{T}\epsilon C_{L}dt\right)}^{2}={\left(\epsilon C_{L}T\right)}^{2}$$

Thus, the error in the final generated image is bounded by $O(\epsilon^{2})$. This quadratic decay implies the system is robust: small linear errors in QFI estimation do not lead to catastrophic divergence in the image generation. □

### Appendix A.3. Proof of Theorem 6

**Proof.** An MPS represents a state vector of size $2^{N}$ as a chain of tensors $A^{\left[i\right]}$ of dimension $\chi \times d_{phys}\times \chi$. The storage cost is $N\cdot d_{phys}\cdot \chi^{2}$. Contraction of a local operator involves updating tensors within the correlation length. The intermediate tensors formed during contraction never exceed dimension $\chi^{2}$ due to SVD truncation. Thus, space complexity is linear in *N* and quadratic in χ, avoiding exponential memory explosion. □
